# PCSK9 promotes T helper 1 and T helper 17 cell differentiation by activating the nuclear factor‐κB pathway in ankylosing spondylitis

**DOI:** 10.1002/iid3.870

**Published:** 2023-05-26

**Authors:** Jianfei Cai, Yinghui Jiang, Fucai Chen, Shubin Wu, Hongjun Ren, Pingping Wang, Jiayong Wang, Wei Liu

**Affiliations:** ^1^ Department of Rheumatology and Immunology Huadong Hospital Affiliated with Fudan University Shanghai China; ^2^ Department of Traditional Chinese Medicine and Pharmacy China Pharmaceutical University Nanjing China; ^3^ Department of Rheumatology and Immunology Shanghai Qiang‐zhi Hospital Shanghai China

**Keywords:** ankylosing spondylitis, nuclear factor‐κB pathway, proprotein convertase subtilisin/kexin type 9, T helper 1 cells, T helper 17 cells

## Abstract

**Objective:**

Our previous study reveals that proprotein convertase subtilisin/kexin type 9 (PCSK9) is positively related to inflammatory markers, T helper (Th)‐17 cells, and treatment response in ankylosing spondylitis (AS) patients. Subsequently, this study aimed to explore the effect of PCSK9 on Th cell differentiation and its potential molecular mechanism in AS.

**Methods:**

Serum PCSK9 was determined by enzyme‐linked immunosorbent assay in 20 AS patients and 20 healthy controls (HCs). Then naïve CD4^+^ T cells were isolated from AS patients and infected with PCSK9 overexpression or knockdown adenovirus followed by polarization assay. Afterward, PMA (an NF‐κB activator) was administrated.

**Results:**

PCSK9 was increased in AS patients compared to HCs (*p* < .001), and it was positively related to Th1 cells (*p* = .050) and Th17 cells (*p* = .039) in AS patients. PCSK9 overexpression increased the CD4^+^IFN‐γ^+^ cells (*p* < .05), CD4^+^IL‐17A^+^ cells (*p* < .01), IFN‐γ (*p* < .01), and IL‐17A (*p* < .01), while it exhibited no effect on CD4^+^IL‐4^+^cells or IL‐4 (both *p* > .05); its knockdown displayed the opposite function on them. Moreover, PCSK9 overexpression upregulated the p‐NF‐κB p65/NF‐κB p65 (*p* < .01), while it had no effect on p‐ERK/ERK or p‐JNK/JNK (both *p* > .05); its knockdown decreased p‐NF‐κB p65/NF‐κB p65 (*p* < .01) and p‐JNK/JNK (*p* < .05). Then, PMA upregulates p‐NF‐κB p65/NF‐κB p65 (*p* < .001) and increased CD4^+^IFN‐γ^+^ cells, CD4^+^IL‐17A^+^ cells, IFN‐γ, and IL‐17A (all *p* < .01), also it alleviated the effect of PCSK9 knockdown on NF‐κB inhibition and Th cell differentiation (all *p* < .01).

**Conclusion:**

PCSK9 enhances Th1 and Th17 cell differentiation in an NF‐κB‐dependent manner in AS, while further validation is necessary.

## Graphical abstract

Our previous study reveals that proprotein convertase subtilisin/kexin type 9 (PCSK9) is positively related to inflammatory markers, T helper (Th)‐17 cells, and treatment response in ankylosing spondylitis (AS) patients. Subsequently, this study aimed to explore the effect of PCSK9 on Th cell differentiation and its potential molecular mechanism in AS. It showed that PCSK9 enhances Th1 and Th17 cell differentiation in an NF‐κB‐dependent manner in AS, while further validation is necessary.

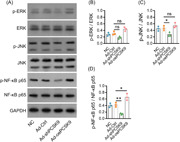

## 1 INTRODUCTION

Ankylosing spondylitis (AS) is an autoimmune disease mainly characterized by inflammation of the axial skeleton and the sacroiliac joints with subsequent bone destruction and erosions.^1^ Although the full landscape of AS pathophysiology remains unclear, some risk factors have been identified as key elements in AS development (such as age, genetic predisposition, immunological dysregulation, etc.).^2^, ^3^ Dysregulated differentiation of T cells has been identified as one critical risk factor for AS etiology and may further impair bone formation.^4^, ^5^ Recently, some mediators have been reported to regulate naïve CD4^+^ T cell differentiation towards T helper (Th)‐1 cells, Th17 cells, or regulatory T (Treg) cells in AS.^6^, ^7^ Hence, identifying more potential inflammatory mediators may unravel the underlying mechanism of AS pathogenesis and serve as a potential therapeutic intervention for AS.

Proprotein convertase subtilisin/kexin type 9 (PCSK9) is responsible for intracellular protease activity and protein trafficking.^8^ Recent studies indicate the potential role of PCSK9 in the differentiation of T cells. For instance, PCSK9 promotes the differentiation of naïve CD4^+^ T cells into Th1 cells and Th17 cells as well as upregulates their secreted cytokines to induce vascular inflammation.^9^, ^10^, ^11^ Furthermore, PCSK9 is implicated in the regulation of the T cell receptor (TCR) signaling pathway, the latter is closely involved in T cell proliferation, activation, and differentiation in various diseases, including AS.^12^, ^13^, ^14^, ^15^ Collectively, these above‐mentioned studies indicate that PCSK9 may contribute to AS pathogenesis.^4^, ^5^

Our previous study reveals that PCSK9 is not only related to AS risk but also correlates with higher C‐reactive protein, Th17 cells, interleukin (IL)‐17A, and better response to tumor necrosis factor (TNF) inhibitor in AS patients.^16^ However, the underlying mechanism of PCSK9 in AS pathogenesis remains unclear. Therefore, the current study aimed to explore the effect of PCSK9 on Th cell differentiation and its potential molecular mechanism in AS.

## 2 METHODS

### 2.1 Human subjects

A total of 20 AS patients and 20 healthy controls (HCs) were recruited from our hospital, and blood samples were acquired from all participants. The level of PCSK9 in the serum was detected with enzyme‐linked immunosorbent assay (ELISA). Peripheral blood mononuclear cells (PBMCs) of AS patients were separated with Ficoll Paque Plus (Univ) and used for detection of Th1/Th2/Th17 cell percentage. The study was approved by the Ethics Committee of Huadong Hospital, Affiliated with Fudan University, with the approval number of 2019K054. The written informed consent were attained from all participants.

### 2.2 Cell isolation and adenovirus infection

Naïve CD4^+^ T cells were isolated from PBMCs of AS patients using an isolation kit (STEMCEL) referring to the kit's instructions, and maintained in RPMI‐1640 medium (Servicebio, China) plus 10% FBS (HyClone) and penicillin/streptomycin solution (Servicebio) at 37°C and 5% CO_2_. The PCSK9 knockdown adenovirus (Ad‐shPCSK9), PCSK9 overexpression adenovirus (Ad‐oePCSK9), and control adenovirus (Ad‐Ctrl) were purchased from Synbio Technologies (Jiangsu, China) and infected the naïve CD4^+^ T cells according to manufacturer's protocols. After 72 h culture, the reverse transcription qPCR (RT‐qPCR), western blot, and Th1/Th2/Th17 cell differentiation assays were performed.

### 2.3 Phorbol‐12‐myristate 13‐acetate (PMA) stimulation

Cells were plated and infected using Ad‐shPCSK9 or Ad‐Ctrl as mentioned above. After 72 h infection, PMA (100 ng/mL; Sigma), an activator of nuclear factor (NF)‐κB,^17^ were added to verify the regulation of PCSK9 on the NF‐κB signaling pathway. After another 24 h stimulation, RT‐qPCR and western blot assays were executed. Furthermore, naïve CD4^+^ T cell differentiation to Th1/Th17 assays were performed in the presence of PMA.

### 2.4 Cell differentiation

For naïve CD4^+^ T cell differentiation, infected cells were cultured in 24‐well plates (2 × 10^5^ cells/well) which were pre‐coated by anti‐CD3 (Abcam) and anti‐CD28 (Abcam), and exposed to different differentiation conditions as previously described.^18^ Briefly, IL‐12 (10 ng/mL; MCE) and anti–IL‐4 (10 μg/mL; Affinity, China) were used for Th1 differentiation. IL‐4 (2.5 ng/mL; MCE) and anti–IFN‐γ (10 μg/mL; Affinity) were added for Th2 differentiation. TGF‐β (5 ng/mL, MCE), IL‐6 (10 ng/mL, MCE), IL‐1β (10 ng/mL, MCE), IL‐23 (20 ng/mL, MCE), anti–IL‐4 and anti–IFN‐γ were added for Th17 differentiation. After 72 h differentiation, cells were harvested for flow cytometry, and the supernatants were used for ELISA.

### 2.5 Flow cytometry and ELISA

The Human Th1/Th2/Th17 phenotyping kit (R&D) was used for detection of the Th1/Th2/Th17 percentages in PBMCs or differentiated naïve CD4^+^ T cells. Briefly, cells were re‐suspended and staining with human anti‐CD4 fluorescent antibody (4°C, 30 min). Next, cells were incubated with Cytofix/Cytoperm buffer (4°C, 30 min), and incubated with human anti‐IFN‐γ, anti‐IL‐4, or anti‐IL‐17A fluorescent antibodies (4°C, 30 min). Lastly, cells were detected using a CytoFlex (Beckman), and results were analyzed by FlowJo software (Ver7.6; FlowJo). The levels of PCSK9, IFN‐γ, IL‐4, or IL‐17A in serum samples and cell supernatants after differentiation were evaluated by ELISA kits (R&D) referring to the kit's instructions.

### 2.6 RT‐qPCR

Total RNA of naïve CD4^+^ T cells was isolated by Beyozol (Beyotime, China). The RT‐qPCR program was completed in the presence of BeyoFast™ RT‐qPCR Kit (Beyotime, China). The level of PCSK9 was calculated by the 2^‐ΔΔCt^ method, which normalized to GAPDH. Primers for PCSK9 and GAPDH were obtained from Sangon (Shanghai, China) and shown as follows (5′–3′): PCSK9 (forward, GCTGAGCTGCTCCAGTTTCT; reverse, AATGGCGTAGACACCCTCAC) and GAPDH (forward, ATGGGGAAGGTGAAGGTCG; reverse, GGGGTCATTGATGGCAACAATA).

### 2.7 Western blot

Naïve CD4^+^ T cells were lysed in protein extraction solution (Solarbio) plus protease and phosphatase inhibitor (Solarbio). Proteins were quantified using BCA reagent (Servicebio). Sodium dodecyl sulfate‐polyacrylamide gel electrophoresis was carried out using precast gel (Willget Biotech), and proteins were transferred onto polyvinylidene fluoride membranes (Servicebio). The membranes were incubated with 5% BSA (37°C, 1.5 h), then incubated with PCSK9 (1:500), p‐ERK (1:1500), ERK (1:3000), p‐JNK (1:1500), JNK (1:3000), p‐NF‐κB p65 (1:1500), NF‐κB p65 (1:3000), or GAPDH antibody (1:8000) (All bought from Abcam; 37°C, 1 h). Afterward, membranes were incubated with secondary antibody (1:5000; 37°C, 1 h; Servicebio). The ECL Kit (Servicebio) was used for detection of blots.

### 2.8 Statistical analysis

Comparison of PCSK9 level between AS patients and HCs was determined by Mann–Whitney *U* test. Correlation of PCSK9 level with Th1/Th2/Th17 percentage in AS patients was assessed with Spearman's rank correlation test. The ANOVA with Tukey's or Dunnett‐t post hoc tests was used for comparison in the cellular experiments. GraphPad Prism (Ver7.0; GraphPad Inc.) was used for all statistical analysis, and *p* < .05 was defined as a significant difference.

## 3 RESULTS

### 3.1 PCSK9 expression and its correlation with Th cells in AS patients

A total of 20 AS patients and 20 HCs were enrolled among whom their mean ages were 37.15 ± 8.84 years and 34.85 ± 8.30 years, respectively. There were 18 (90%) males and 2 (10%) females in AS patients; 16 (80%) males and 4 (20%) females in HCs. Besides, the mean Bath Ankylosing Spondylitis Disease Activity Index (BASDAI) of AS patients was 5.90 ± 1.45.

**Figure 1:**
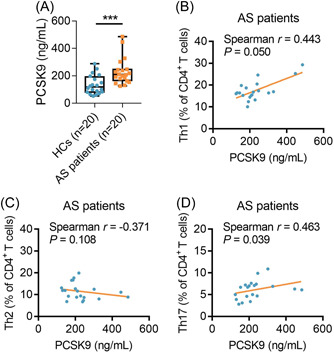
PCSK9 was positively related to Th1 cells and Th17 cells in AS patients. Comparison of PCSK9 level between AS patients and HCs (A). Correlation of PCSK9 level with Th1 cells (B), Th2 cells (C), and Th17 cells (D) in AS patients. AS, ankylosing spondylitis; HCs, healthy controls; PCSK9, proprotein convertase subtilisin/kexin type 9.

The PCSK9 level was increased in AS patients compared to HCs (*p* < .001, Figure 1A). Furthermore, the PCSK9 level was positively related to Th1 cells (*r* = .443, *p* = .050) and Th17 cells (*r* = .463, *p* = .039) in AS patients, while it did not associate with Th2 cells (*r* = −0.371, *p* = .108) (Figure 1B–D).

### 3.2 Effect of PCSK9 on Th cell differentiation in AS

**Figure 2:**
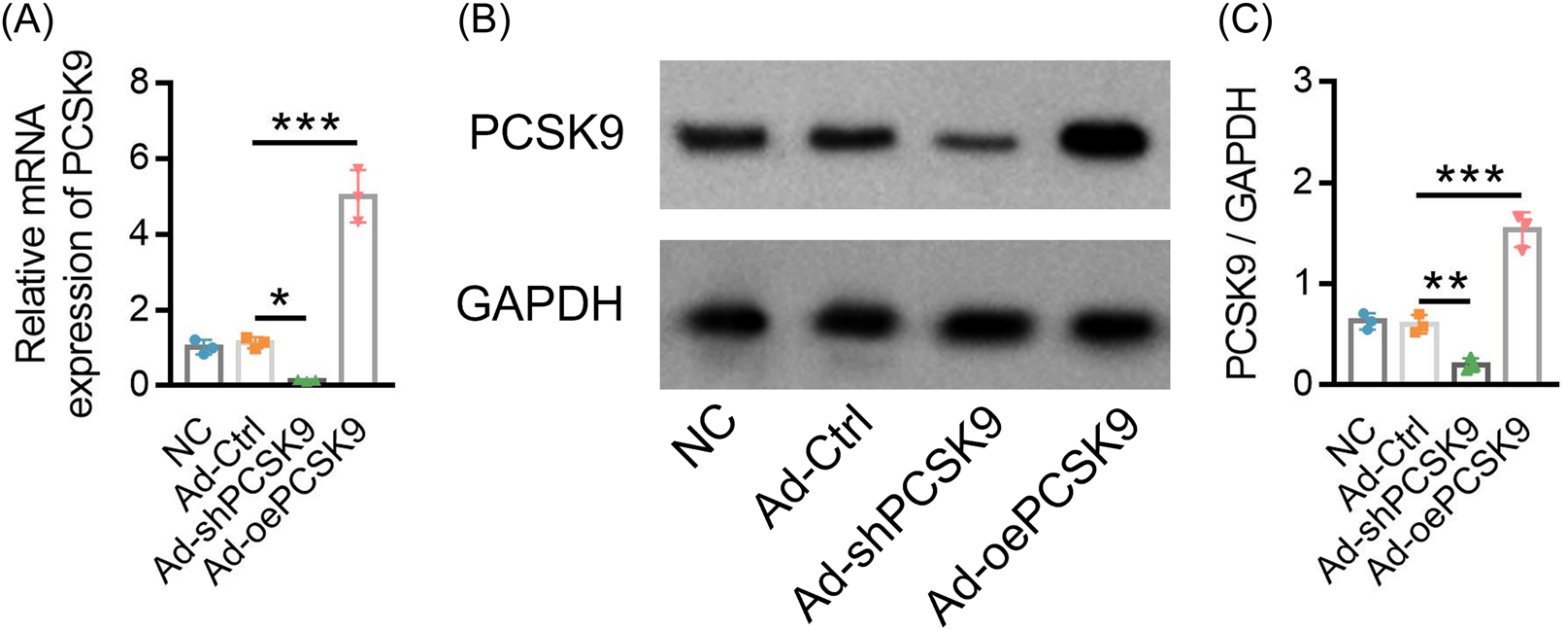
Assessment of transfection efficiency. Comparison of PCSK9 relative mRNA expression among NC, Ad‐Ctrl, Ad‐shPCSK9, and Ad‐oePCSK9 groups (A). Presentation of western blot bands (B) and further comparison of PCSK9 protein expression among NC, Ad‐Ctrl, Ad‐shPCSK9, and Ad‐oePCSK9 groups (C). The naïve CD4^+^ T cells, which were isolated from PBMCs of AS patients, were applied for the assessment of transfection efficiency. The sample number in each group was 3. The ANOVA with Tukey's or Dunnett‐t post hoc tests for comparison was applied. ANOVA, analysis of variance; AS, ankylosing spondylitis; mRNA, messenger RNA; PCSK9, proprotein convertase subtilisin/kexin type 9.

Ad‐oePCSK9 increased PCSK9 mRNA expression (*p* < .001; Figure 2A) and PCSK9 protein expression (*p* < .001; Figure 2B,C). However, Ad‐shPCSK9 decreased PCSK9 mRNA expression (*p* < .05) and PCSK9 protein expression (*p* < .01; Figure 2A–C), suggesting successful transfection.

**Figure 3:**
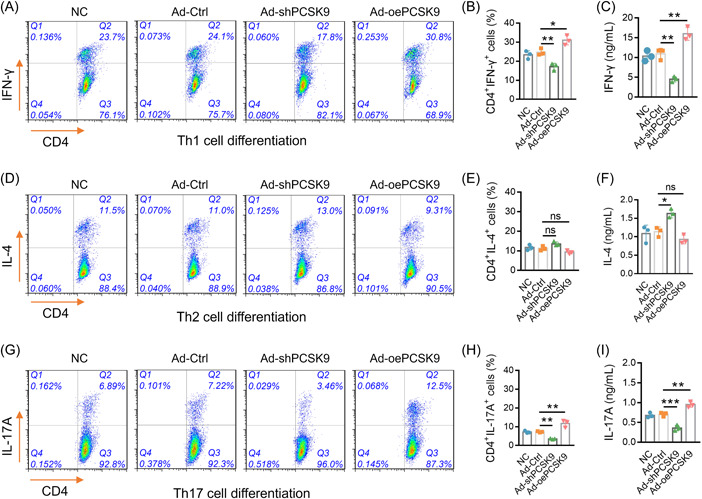
PCSK9 enhanced Th1 and Th17 cell differentiation. Presentation of flow cytometry images of CD4^+^IFN‐γ^+^ cells (A). Comparison of CD4^+^IFN‐γ^+^ cells (B) and IFN‐γ levels (C) among NC, Ad‐Ctrl, Ad‐shPCSK9, and Ad‐oePCSK9 groups. Presentation of flow cytometry images of CD4^+^IL‐4^+^ cells (D). Comparison of CD4^+^IL‐4^+^ cells (E) and IL‐4 levels (F) among NC, Ad‐Ctrl, Ad‐shPCSK9, and Ad‐oePCSK9 groups. Presentation of flow cytometry images of CD4^+^IL‐17A^+^ cells (G). Comparison of CD4^+^IL‐17A^+^ cells (H) and IL‐17A levels (I) among NC, Ad‐Ctrl, Ad‐shPCSK9, and Ad‐oePCSK9 groups. The sample number in each group was 3. The ANOVA with Tukey's or Dunnett‐t post hoc tests for comparison was applied. ANOVA, analysis of variance; IFN‐γ, interferon‐gamma; IL, interleukin; PCSK9, proprotein convertase subtilisin/kexin type 9.

After the transfection, the flow cytometry assay and ELISA were carried out to explore the effect of PCSK9 on Th cell differentiation. Then, it was observed that Ad‐oePCSK9 enhanced CD4^+^IFN‐γ^+^ cells (*p* < .05; Figure 3A,B) and IFN‐γ level (*p* < .01; Figure 3C), while it did not alter CD4^+^IL‐4^+^ cells (*p* > .05; Figure 3D,E) OR IL‐4 level (*p* > .05; Figure 3F). Moreover, Ad‐oePCSK9 raised CD4^+^IL‐17A^+^ cells (*p* < .01; Figure 3G,H) AND IL‐17A level (*p* < .01; Figure 3I) Furthermore, Ad‐shPCSK9 displayed an opposite function as that of Ad‐oePCSK9 except that Ad‐shPCSK9 also upregulated IL‐4 (Figure 3A–I). These data suggested that PCSK9 promoted Th1 and Th17 cell differentiation, while it was less involved in Th2 differentiation.

### 3.3 Regulatory function of PCSK9 on TCR pathway in AS

**Figure 4:**
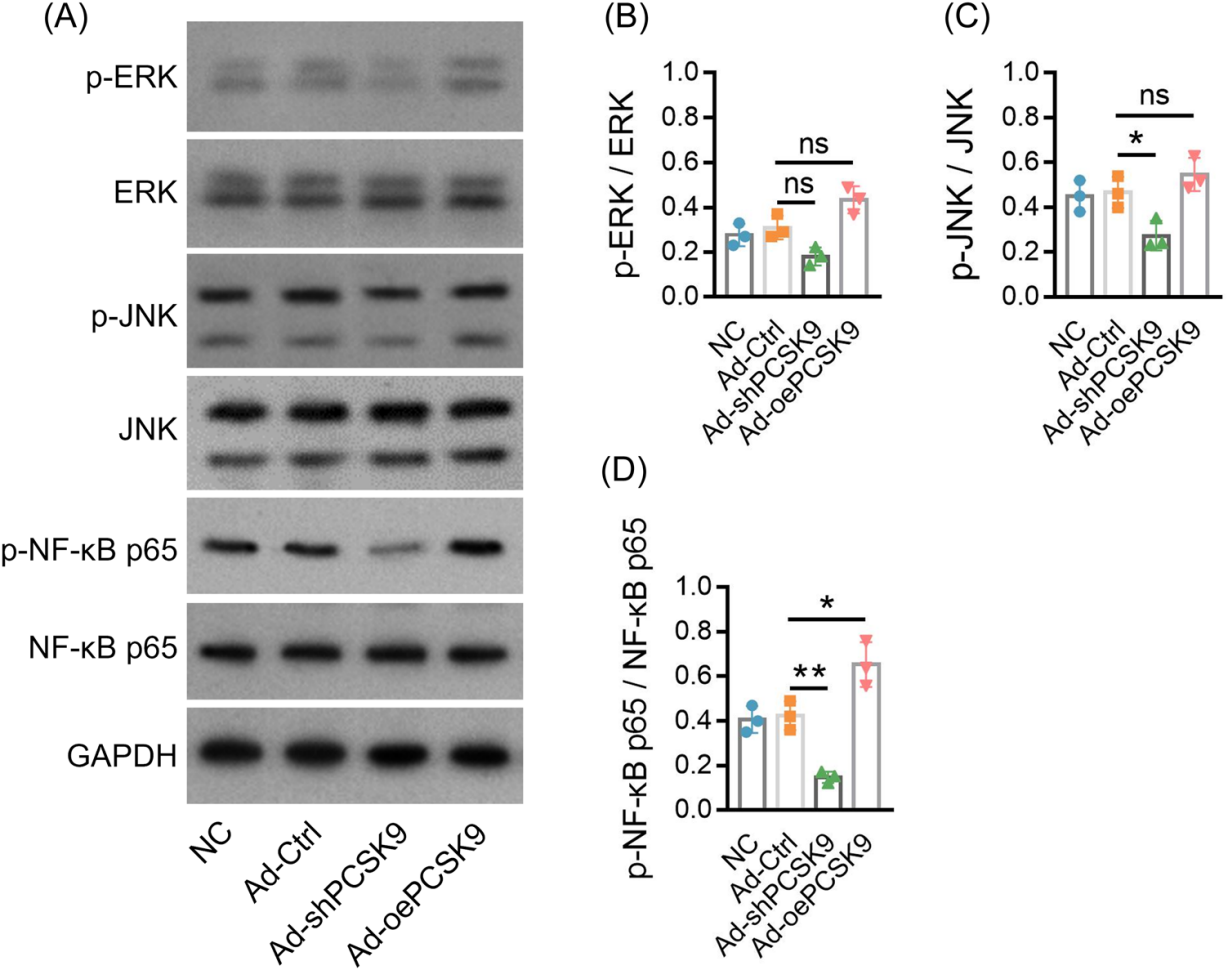
PCSK9 activated the NF‐κB pathway. Presentation of western blot bands (A). Comparison of p‐ERK/ERK (B), p‐JNK/JNK (C), and p‐NF‐κB p65/NF‐κB p65 (D) among NC, Ad‐Ctrl, Ad‐shPCSK9, and Ad‐oePCSK9 groups. The sample number in each group was 3. The ANOVA with Tukey's or Dunnett‐t post hoc tests for comparison was applied. ANOVA, analysis of variance; NF‐κB, nuclear factor‐κB pathway; PCSK9, proprotein convertase subtilisin/kexin type 9.

Previous studies indicate that PCSK9 is involved in the ERK pathway, JNK pathway, and NF‐κB pathway in inflammation‐mediated disease.^19^, ^20^, ^21^ Thus, the current study detected these above‐mentioned pathways through western blot assay and revealed that Ad‐oePCSK9 did not change the expression of p‐ERK/ERK or p‐JNK/JNK (both *p* > .05; Figure 4A–C). Moreover, Ad‐shPCSK9 exhibited no effect on p‐ERK/ERK (*p* > .05), while it decreased p‐JNK/JNK (*p* < .05; Figure 4A–C). Importantly, Ad‐oePCSK9 upregulated p‐NF‐κB p65/NF‐κB p65 (*p* < .05), while Ad‐shPCSK9 downregulated p‐NF‐κB p65/NF‐κB p65 (*p* < .01; Figure 4D). These data suggested that PCSK9 activated the NF‐κB pathway in AS.

### 3.4 Effect of PMA on PCSK9‐induced Th cell differentiation in AS

**Figure 5:**
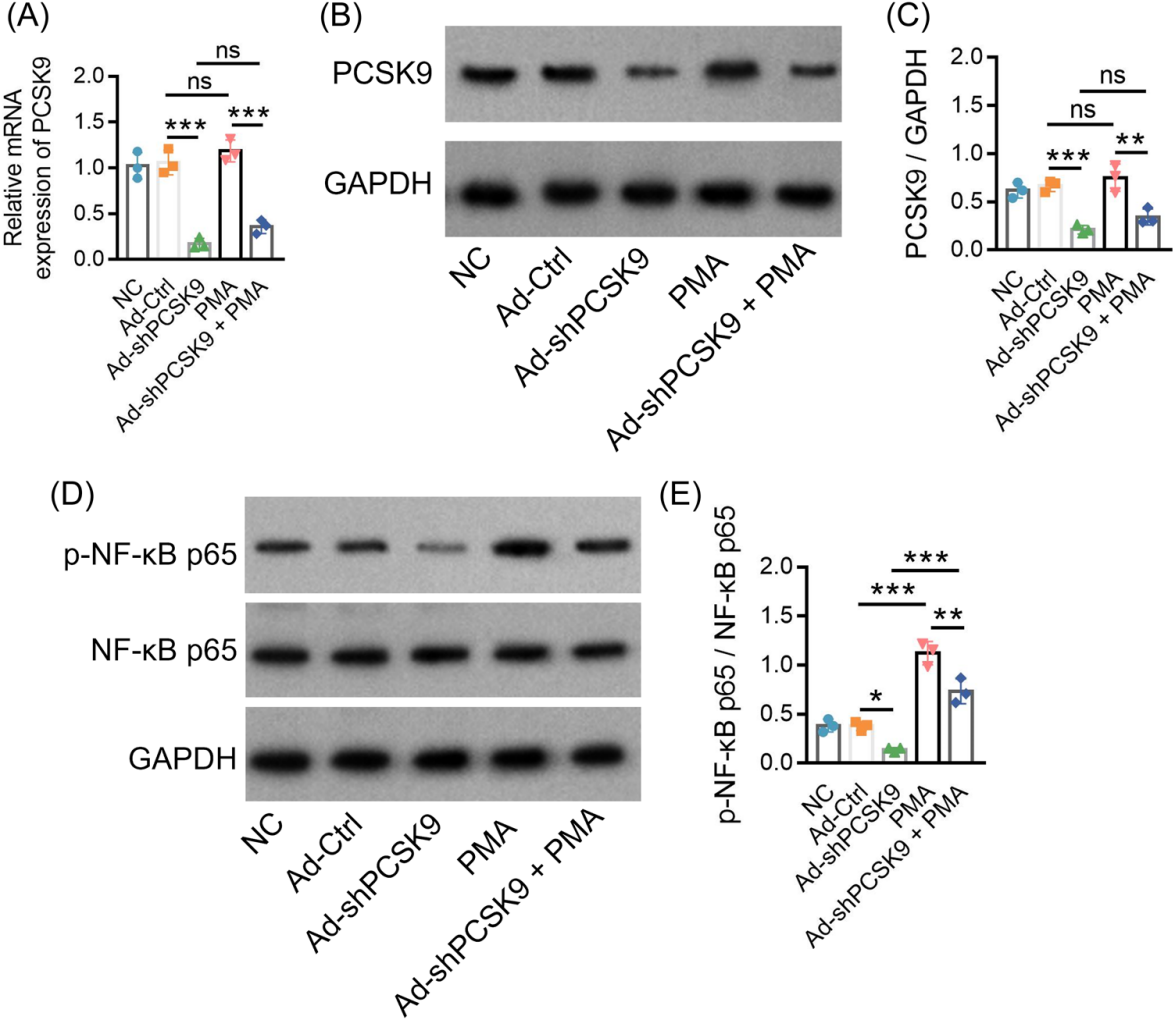
PMA attenuated the effect of Ad‐shPCSK9 on NF‐κB activation. Comparison of PCSK9 relative mRNA expression among NC, Ad‐Ctrl, Ad‐shPCSK9, PMA, and Ad‐shPCSK9 + PMA groups (A). Presentation of western blot bands (B) and further comparison of PCSK9 protein expression among NC, Ad‐Ctrl, Ad‐shPCSK9, PMA, and Ad‐shPCSK9 + PMA groups (C). Presentation of western blot bands (D) and further comparison of p‐NF‐κB p65/NF‐κB p65 (E) among NC, Ad‐Ctrl, Ad‐shPCSK9, PMA, and Ad‐shPCSK9 + PMA groups. The sample number in each group was 3. The ANOVA with Tukey's or Dunnett‐t post hoc tests for comparison was applied. ANOVA, analysis of variance; mRNA, messenger RNA; NF‐κB, nuclear factor‐κB pathway; PMA, phorbol‐12‐myristate 13‐acetate; PCSK9, proprotein convertase subtilisin/kexin type 9.

Then, PMA (an activator of NF‐κB) was added into naïve CD4^+^ T cells that were infected with Ad‐shPCSK9 and Ad‐Ctrl followed by polarization assay. Then, it was noticed that PMA did not alter the PCSK9 mRNA expression (Figure 5A) or PCSK9 protein expression (both *p* > .05) (Figure 5B,C). Moreover, PMA upregulated p‐NF‐κB p65/NF‐κB p65 expression, and it attenuated the effect of Ad‐shPCSK9 on NF‐κB inactivation as well (both *p* < .001, Figure 5D,E).

**Figure 6:**
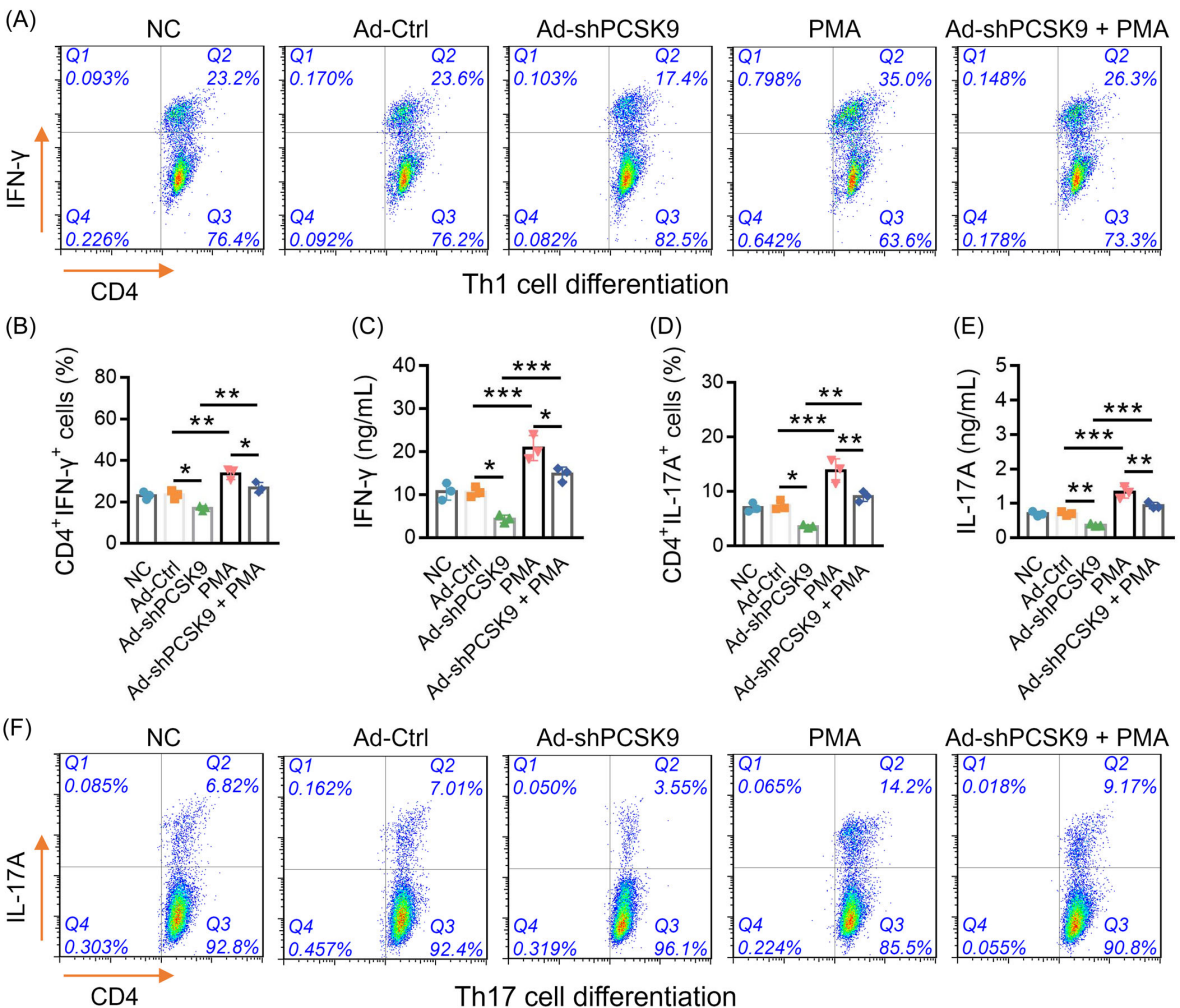
PMA alleviated the effect of Ad‐shPCSK9 on Th1 and Th17 cell differentiation. Presentation of flow cytometry images of CD4^+^IFN‐γ^+^ cells (A). Comparison of CD4^+^IFN‐γ^+^ cells (B) IFN‐γ levels (C) and among NC, Ad‐Ctrl, Ad‐shPCSK9, PMA, and Ad‐shPCSK9 + PMA groups. Presentation of flow cytometry images of CD4^+^IL‐17A^+^ cells (D). Comparison of IL‐17A levels (E) and CD4^+^IL‐17A^+^ cells (F) among NC, Ad‐Ctrl, Ad‐shPCSK9, PMA, and Ad‐shPCSK9+PMA groups. The sample number in each group was 3. The ANOVA with Tukey's or Dunnett‐t post hoc tests for comparison was applied. ANOVA, analysis of variance; IFN‐γ, interferon‐gamma; PMA, phorbol‐12‐myristate 13‐acetate; PCSK9, proprotein convertase subtilisin/kexin type 9.

Moreover, PMA increased CD4^+^IFN‐γ^+^cells (*p* < .01) and IFN‐γ level (*p* < .001) (Figure 6A–C), suggesting its promotive function on Th1 differentiation. Besides, PMA enhanced CD4^+^IL‐17A^+^cells and IL‐17A level (both *p* < .001) (Figure 6D–F), suggesting it could enhance the Th17 differentiation. Furthermore, PMA also alleviated the effect of Ad‐shPCSK9 on Th1 and Th17 cell differentiation. These data indicated that PCSK9 may promote Th1 and Th17 cell differentiation through the activation of the NF‐κB pathway in AS.

## 4 DISCUSSION

The clinical role of PCSK9 in autoimmune diseases has previously been reported. For instance, serum PCSK9 level is increased in systemic lupus erythematosus (SLE) patients compared to HCs, and it is positively related to disease activity and inflammatory markers in SLE patients.^22^ Moreover, another study reports that lower PCSK9 at baseline and at 12 months after TNF inhibitor therapy is related to better response in rheumatoid arthritis (RA) patients.^23^ In the current study, it was revealed that PCSK9 was upregulated in AS patients compared to HCs. The possible reason for these findings was that: PCSK9 was involved in the regulation of T‐cell differentiation, inflammatory cytokines, and the subsequent bone erosions, where the latter three factors were the hallmarks of AS; thus PCSK9 was elevated in AS patients compared with HCs.^2^, ^9^, ^24^, ^25^ Moreover, it was also observed that PCSK9 was positively related to the Th1 cells and Th17 cells in AS patients from the current study, and the reason for this finding was that PCSK9 might mediate multiple pathways (such as NF‐κB pathway, ERK pathway, etc.) to influence the Th1 and Th17 cell differentiation.^19^, ^20^

T‐cell differentiation plays a critical role in AS pathophysiology. Recent studies display that Th17 cell polarization is enhanced in AS through genome sequencing studies.^26^, ^27^ Moreover, increased Th17 cells further upregulate the secretion of inflammatory cytokines (such as IL‐17, TNF‐α, IFN‐γ, etc.) and induce an inflammatory response to active osteoclasts for bone destruction in AS.^28^ In terms of the regulatory function of PCSK9 on T cell differentiation, several previous studies indicate that PCSK9 promotes Th1 and Th17 cell differentiation while inhibiting Treg differentiation.^9^, ^10^, ^11^ Considering the critical role of PCSK9 on T cell differentiation, it is reasonable to hypothesize that PCSK9 may regulate T cell differentiation in AS as well, while the relevant study is limited. Hence, the present study collected naïve CD4^+^ T cells from AS patients and infected them with PCSK9 overexpression or knockdown adenovirus followed by polarization array. Then, it was observed that PCSK9 enhanced Th1 and Th17 cell differentiation and their secreted cytokines in AS. A possible reason was applied to explain this finding: PCSK9 might target several signaling pathways (such as NF‐κB pathways, ERK pathways, etc.) and transcription factors (such as RORC, SOCS1, etc.) to induce the Th1 and Th17 differentiation in AS.^9^, ^10^, ^19^, ^20^ However, further in vivo studies could be conducted to further validate the impact of PCSK9 in AS. Recently, it should be noticed that other cell types, such as mesenchymal stem cells, osteoblast, and osteoclast, might crosstalk with the immune cells such as T cells, B cells, etc. to further involve in the pathogenesis of AS through the osteoimmunological pathogenesis of AS.^29^ Furthermore, PCSK9 is also reported to act as a role in inhibiting the osteoclasts.^30^ Hence, it was reasonable to hypothesize that PCSK9 might also take part in the pathogenesis of AS by regulating other cells (such as osteoclasts). However, this hypothesis still needed further validation.

The TCR signaling network represents multiple complicated signaling pathways that are responsible for the activation, proliferation, and differentiation of T cells.^12^, ^31^, ^32^ Previous studies indicate that PCSK9 is involved in regulating ERK, JNK, and NF‐κB pathways in different diseases, and these pathways are known to be part of the TCR signaling network.^12^, ^19^, ^20^, ^21^ Therefore, it is reasonable to hypothesize that PCSK9 may also act on ERK, JNK, and NF‐κB pathways in AS, while the relevant study is limited. Hence, the present study revealed that PCSK9 only acted on the NF‐κB pathway instead of the ERK or JNK pathway in AS. Then, PMA (an NF‐κB activator) was administrated for further experiments, and it was observed that PMA administration enhanced the Th1 and Th17 cell differentiation in AS, which could be attributed to the promotive function of PMA on the NF‐κB pathway to induce the Th cell differentiation.^31^, ^33^ Moreover, PMA attenuated the influence of PCSK9 knockdown on Th cell differentiation in AS. These data suggest that the NF‐κB pathway might be deeply implicated in the regulation of PCSK9 on Th cell differentiation in AS. Interestingly, another previous study applies tetradecanoylphorbol‐13‐acetate (TPA) for the inhibition of the NF‐κB pathway activation, and they get a more satisfying finding, which might imply that the TPA might be more suitable for the inhibition of NF‐κB pathway activation. Hence, other specific NF‐κB pathway antagonists could be applied in the following studies to verify the effect of NF‐κB modification on the regulation of PCSK9 in AS pathogenesis (such as TPA).^34^

Several limitations in our study were non‐neglectable: (1) The lack of a further in vivo study for validation might reduce the reliability of our study, which could be conducted in the future; (2) Even though a rescue experiment was designed in the current study, only PMA but not other NF‐κB pathway antagonists was applied in our study; further studies could apply other NF‐κB pathway antagonists and/or agonists for a validation (such as TPA).

In conclusion, PCSK9 promotes Th1 and Th17 cell differentiation by activating the NF‐κB pathway in AS, while further validation is warranted.

## AUTHOR CONTRIBUTIONS

**Jianfei Cai**: Data curation; formal analysis. **Yinghui Jiang**: Data curation; formal analysis; investigation. **Fucai Chen**: Formal analysis; investigation; resources. **Shubin Wu**: Data curation; formal analysis; investigation. **Hongjun Ren**: Resources; visualization; writing—original draft. **Pingping Wang**: Formal analysis; investigation; methodology; writing—original draft. **Jiayong Wang**: Formal analysis; methodology; writing—original draft. **Wei Liu**: Conceptualization; resources; supervision; validation; writing—review & editing.

## CONFLICTS OF INTEREST STATEMENT

The authors declare no conflicts of interest.

## Data Availability

The datasets generated during and/or analyzed during the current study are available from the corresponding author on reasonable request.
